# The Influence of Social Media Use on Waste Sorting Intentions: A Cognition–Affect–Conation Model Integration with Social Amplification of Risk Framework

**DOI:** 10.3390/bs16020305

**Published:** 2026-02-21

**Authors:** Yixin Chen, Huiting Tang, Ying Lian

**Affiliations:** 1 School of Marxism Studies, University of Science and Technology Beijing, 30 Xueyuan Road, Haidian District, Beijing 100083, China; 2 School of Journalism, Communication University of China, No. 1 Dingfuzhuang East Street, Beijing 100024, China

**Keywords:** social media use, waste sorting behavior, cognition-affect-conation model, social amplification of risk framework, behavioral intentions

## Abstract

This study examines the impact of social media use on public behavioral intentions regarding waste sorting in China, integrating the Cognition–Affect–Conation model with the Social Amplification of Risk Framework. The proposed framework explores how social media exposure and gratification influence waste sorting intentions through anticipated emotions and environmental risk perception. Regression analysis confirms that information gratification primarily activates positive emotions, while information exposure has a stronger effect on negative emotions. Both affective pathways significantly predict waste sorting intentions, with comparable predictive strengths. Mediation analysis further reveals that information gratification and information exposure indirectly influence behavioral intention through dual emotional pathways and environmental risk perception. Qualitative interviews highlight two structural deficiencies: fragmented knowledge dissemination, which weakens environmental norm internalization, and uneven community integration, which limits behavioral translation. These findings underscore the need for diversified communication strategies and community-based policy interventions to enhance public participation in waste sorting.

## 1. Introduction

Waste sorting is a cornerstone of sustainable urban development, directly contributing to resource conservation, environmental protection, and the advancement of circular economies (Yang et al., 2021). The successful implementation of urban waste sorting policies depends not only on robust governmental measures but also on the active engagement and deep participation of the public (Tian et al., 2022). Thus, a pressing challenge lies in how to stimulate public enthusiasm for waste sorting, identify the factors influencing their willingness to participate, and enhance their motivation to engage in waste sorting initiatives.

Existing research has predominantly examined the relationship between behavioral intentions and current or future waste-sorting behaviors or participation levels (Ho et al., 2015; Negash et al., 2021). A growing body of empirical work further suggests that perceived risks and perceived benefits play critical roles in shaping individuals’ waste-sorting intentions (Sengupta et al., 2021; Veliverronena & Davidsone, 2024). While these studies offer important insights into behavioral determinants, they tend to focus primarily on outcome-oriented predictors, with comparatively less attention paid to the communicative environments and affective processes through which such intentions are formed, interpreted, and sustained.

In particular, although recent studies have shown that media use can promote pro-environmental behaviors (Han et al., 2025; Huang et al., 2023), existing research often treats media exposure as a homogeneous construct and overlooks the differentiated roles of information exposure and information gratification in shaping environmental engagement. Moreover, emotional and psychological processes—such as anticipated emotional responses and perceptions of environmental risk—are frequently incorporated as abstract mediators in quantitative models, while the subjective meanings and contextual interpretations underlying these processes remain underexplored. This gap suggests that survey-based analyses alone may be insufficient to fully capture how individuals interpret waste-sorting information and translate emotional reactions into behavioral intentions.

From a theoretical perspective, the Cognition–Affect–Conation (CAC) model highlights the sequential relationship between cognitive evaluation, emotional response, and behavioral intention (Lyu et al., 2024). Complementarily, the Social Amplification of Risk Framework (SARF) emphasizes the role of media in shaping risk perceptions, eliciting emotional reactions, and amplifying social responses through information transmission processes (Lu & Xiao, 2024). Media characteristics such as information volume, framing, and emotional tone can substantially influence how environmental risks are perceived and emotionally processed (Lee et al., 2023; Zhang et al., 2025). However, when applied independently, CAC primarily captures internal psychological processes, while SARF focuses on risk communication dynamics without fully explicating how these processes translate into individual behavioral intentions. This study advances existing research by explicitly integrating CAC and SARF to bridge media-driven risk communication with individual-level affective mechanisms. By doing so, it conceptualizes social media as a critical interface through which external risk information is cognitively evaluated, emotionally processed, and ultimately transformed into behavioral intention. This integration allows for a more fine-grained understanding of how differentiated forms of social media use activate distinct emotional pathways and shape waste-sorting intentions.

Building on these theoretical foundations, this study examines waste-sorting information as a communicative context to investigate how social media use influences public waste-sorting intentions. Specifically, grounded in the CAC model and informed by SARF, we develop a conceptual framework in which social media use (information exposure and information gratification) affects anticipated positive and negative emotions and environmental risk perception, which in turn shape waste-sorting intentions. Given the inherently interpretive nature of emotional and risk-related processes, this study adopts a mixed-methods research design. A questionnaire survey is employed to systematically test the proposed relationships, while semi-structured interviews are used to provide contextual and explanatory insights into how individuals perceive waste-sorting information, experience emotional reactions, and interpret environmental risks in everyday contexts. By integrating quantitative and qualitative evidence, this study aims to offer a more nuanced and theoretically grounded understanding of the mechanisms through which social media use shapes waste-sorting intentions.

## 2. Theoretical Foundation and Hypothesis Development

### 2.1. Social Media Use and Waste-Sorting Intentions

A substantial body of research on pro-environmental behavior conceptualizes behavioral intention as a proximal antecedent of actual environmental practices, including waste sorting (Ho et al., 2015; Negash et al., 2021). Within this literature, media environments are increasingly recognized as critical contextual factors that shape intention formation by structuring individuals’ access to information, perceived importance of environmental issues, and awareness of behavioral consequences.

With the widespread adoption of social media, these platforms have become primary channels through which the public encounters waste-sorting-related information, policy messages, and environmental narratives. Empirical evidence suggests that social media use can directly promote pro-environmental intentions by increasing issue salience and facilitating information acquisition. Prior studies focusing on waste sorting have shown that higher levels of social media use are significantly associated with stronger voluntary waste-sorting intentions, largely by enhancing individuals’ environmental knowledge and the perceived importance of waste sorting (Ai et al., 2021).

Importantly, prior studies indicate that social media use is not a unitary construct. Drawing on the Uses and Gratifications Theory, scholars distinguish between information exposure, which captures the frequency and intensity with which individuals encounter relevant information, and information gratification, which reflects the extent to which such information is perceived as useful, understandable, and satisfying (Abid & Harrigan, 2020). These two dimensions imply distinct psychological mechanisms.

On the one hand, information exposure increases the likelihood of repeated contact with waste-sorting rules, policy requirements, and environmental consequences. Research in communication and behavioral science has long shown that repeated media exposure enhances issue salience and information accessibility, thereby shaping individuals’ judgments and behavioral intentions (McCombs, 2005). From a behavioral perspective, increased exposure to relevant information strengthens the cognitive basis for intention formation by making behavioral considerations more salient and easier to retrieve (Ajzen, 2020). In environmental contexts, scholars have further emphasized that frequent exposure to environmental information contributes to greater awareness and concern, which are important antecedents of pro-environmental engagement (Kollmuss & Agyeman, 2002). Together, these insights suggest that repeated exposure to waste-sorting-related information via social media can increase cognitive salience and provide a foundation for stronger waste-sorting intentions.

On the other hand, information gratification represents a more active and in-depth mode of media use. When individuals perceive social media content as helpful for understanding environmental problems, clarifying behavioral procedures, and interpreting policy logic, they are more likely to develop a sense of competence and behavioral efficacy. According to the Uses and Gratifications Theory, gratification-oriented media use reflects purposeful information seeking aimed at satisfying cognitive and instrumental needs, which in turn facilitates deeper information processing and internalization (Katz et al., 1973). From a behavioral perspective, such cognitively rewarding information use strengthens perceived behavioral control and perceived usefulness, both of which are key antecedents of behavioral intention formation (Ajzen, 2020; Davis, 1989). In pro-environmental contexts, prior research has consistently shown that informational satisfaction and perceived usefulness derived from environmental communication are positively associated with stronger environmental attitudes and intentions (Steg & Vlek, 2009). Accordingly, gratification-based social media use is more likely to promote waste-sorting intentions by enhancing individuals’ understanding, perceived competence, and perceived importance of the behavior, rather than through passive information exposure alone.

Taken together, these findings suggest that both frequent exposure to waste-sorting information and gratification-oriented information use can directly enhance waste-sorting intentions, albeit through different informational mechanisms. Accordingly, this study proposes the following hypotheses:

**H1a.**  *Information exposure from social media use positively influences waste-sorting intentions.*

**H1b.**  *Information gratification from social media use positively influences waste-sorting intentions.*

### 2.2. Affective Pathways of Anticipated Emotions in the CAC Model

Beyond direct informational effects, pro-environmental intentions are also shaped by affective processes. The CAC model posits that cognitive evaluations of information often elicit emotional responses, which subsequently motivate behavioral intentions (Bagozzi et al., 1999). In media-saturated environments, individuals’ emotional reactions to environmental issues are frequently generated through mediated information rather than direct experience.

Anticipated emotions refer to the emotions individuals expect to experience as a result of engaging in, or failing to engage in, a specific behavior. In the context of waste sorting, anticipated positive emotions may include pride, satisfaction, and a sense of meaningful contribution, whereas anticipated negative emotions may involve guilt, regret, or anxiety associated with non-compliance. Prior research has shown that both types of anticipated emotions can motivate pro-environmental intentions, although they operate through different motivational mechanisms (Ferguson & Branscombe, 2010; Gao et al., 2024).

Social media use is a particularly important trigger of anticipated emotions, yet different dimensions of use are likely to activate distinct emotional pathways (Berger & Milkman, 2012). Information gratification is more closely associated with anticipated positive emotions. According to the Uses and Gratifications Theory, when individuals perceive that their informational needs are effectively satisfied, they are more likely to experience positive affective responses such as pleasure, satisfaction, and a sense of competence (Locke, 1997; Sundar & Singh, 2013). In the context of waste-sorting communication, content that clearly explains sorting procedures, policy rationales, and environmental benefits can reduce uncertainty and enhance individuals’ perceived behavioral efficacy, thereby strengthening anticipated pride and a sense of meaning associated with compliant behavior (Skokov, 2022). Moreover, the interactive and participatory affordances of social media, often embedded in gratification-oriented use, have been shown to intensify emotional engagement and user involvement compared with passive information reception, as interactive cues increase perceived agency and psychological immersion (Sundar & Singh, 2013).

In contrast, information exposure is more likely to activate anticipated negative emotions. Environmental issues on social media are frequently framed in terms of risks, threats, and adverse consequences, and repeated exposure to such content can heighten emotional responses such as concern, guilt, or anxiety. Empirical studies indicate that negative environmental information spreads more rapidly and widely on social media, increasing the likelihood that frequent exposure will elicit anticipated negative emotions related to behavioral inaction. Accordingly, this study proposes:

**H2a.**  *Information gratification is a stronger predictor of anticipated positive emotions than information exposure.*

**H2b.**  *Information exposure is a stronger predictor of anticipated negative emotions than information gratification.*

Anticipated emotions are then expected to translate into behavioral intentions. Anticipated positive emotions function as reward expectations that reinforce willingness to act, whereas anticipated negative emotions operate as avoidance motivations that encourage individuals to prevent emotional discomfort associated with non-compliance. Prior studies grounded in fear-appeal and emotion-based persuasion models suggest that both positive and negative anticipated emotions can promote adaptive, protective behaviors. Thus, the following hypotheses are proposed:

**H3a.**  *Anticipated positive emotions positively influence waste-sorting intentions.*

**H3b.**  *Anticipated negative emotions positively influence waste-sorting intentions.*

### 2.3. Cognitive Pathways of Environmental Risk Perception in the SARF

In addition to affective mechanisms, cognitive evaluations of environmental risk constitute a central pathway linking social media use to pro-environmental intentions. The SARF conceptualizes media systems as key “amplification stations” that shape public risk perceptions through information volume, repetition, framing, and social interaction (Kasperson et al., 1988).

A substantial body of research has demonstrated that environmental risk perception is a significant predictor of pro-environmental intentions and behaviors. When individuals perceive environmental problems as severe, salient, and personally relevant, they are more likely to support and intend to engage in protective actions (Slovic, 2020). This relationship has been consistently observed across environmental domains, including pollution control, climate change mitigation, and resource conservation.

Importantly, risk perception is not formed in isolation but is socially and medially constructed. Media exposure plays a crucial role in shaping how environmental risks are interpreted, evaluated, and prioritized by the public. Prior research shows that increased media attention and repeated exposure to environmental risk information heighten perceived severity and urgency, thereby strengthening pro-environmental concern and behavioral intentions (Wakefield & Elliott, 2003).

Social media further intensifies these amplification processes by increasing the visibility, vividness, and emotional framing of environmental risks through images, narratives, and interactive discussion. Compared with traditional media, social media enable rapid circulation of risk-related content and peer-to-peer reinforcement, which can magnify perceived environmental threats and their social relevance (O’Neill & Nicholson-Cole, 2009).

From this perspective, environmental risk perception meets the criteria of a mediating variable: it is shaped by social media use and, in turn, influences waste-sorting intentions. Accordingly, this study proposes:

**H4a.**  *Information exposure from social media use positively influences environmental risk perception.*

**H4b.**  *Information gratification from social media use positively influences environmental risk perception.*

**H5.**  *Environmental risk perception mediates the relationship between social media use and waste-sorting intentions.*

## 3. Research Design

### 3.1. Key Variables

In this study, the independent variable is social media use, which is operationalized along two dimensions: information exposure (IE) and information gratification (IG). The mediating variables include anticipated emotions, distinguished as anticipated positive emotions (APE) and anticipated negative emotions (ANE), as well as environmental risk perception (ERP). The dependent variable is waste-sorting intentions (WSI), referring to individuals’ willingness to engage in waste-sorting practices.

Based on the literature review, this study integrates the “information–media–individual” communication mechanism from the SARF with the CAC model to examine the mechanisms through which social media use influences public waste-sorting intentions. The definitions, operationalizations, and corresponding variable codes are summarized in Table 1, and these abbreviations (IE, IG, APE, ANE, ERP, and WSI) are used consistently throughout the subsequent analyses.

To not only examine the statistical relationships among these variables but also to better understand how individuals interpret waste-sorting information and emotional experiences in real-life contexts, this study adopts a mixed-methods research design. Specifically, a questionnaire survey is used as the primary method to test the proposed relationships, while qualitative interviews are incorporated as a complementary approach to provide contextual and explanatory insights into the quantitative findings.

To investigate the impact of social media use on waste-sorting intentions, this study introduces anticipated emotions (both positive and negative) as variables within the internal influence pathway. Based on a synthesis of relevant research, this study operationally defines social media use, anticipated emotions, and waste-sorting intentions. By modifying and refining measurement indicators, while controlling for other variables, a questionnaire was designed to facilitate the investigation. The research aims to better understand the mechanisms through which social media influences waste-sorting intentions, providing a scientific basis for the formulation and implementation of environmental protection policies.

#### 3.1.1. Dependent Variable: Waste-Sorting Intentions

Waste-sorting intentions serve as a key indicator for assessing public behavioral tendencies toward waste disposal. Consistent with the Theory of Planned Behavior, behavioral intention is widely regarded as the most proximal predictor of actual behavior (Ajzen, 2020). By examining individuals’ intentions and attitudinal orientations, this study seeks to capture their underlying propensity to engage in waste-sorting practices and broader pro-environmental behavior. As shown in Table 2, the measurement items were adapted from established scales used in prior research on pro-environmental and waste-related behavioral intentions. All items were measured using a five-point Likert scale to ensure measurement reliability and comparability.

#### 3.1.2. Independent Variable: Social Media Use

The independent variable, social media use, captures individuals’ engagement with waste-sorting-related information on social media, including both the frequency of information exposure and the perceived usefulness of such content. The operationalization of this construct was adapted from the Facebook Intensity Scale, with items modified to reflect the waste-sorting context (Skokov, 2022). The specific measurement items for information exposure and information gratification are presented in Table 3.

#### 3.1.3. Mediating Variable: Anticipated Emotions and Environmental Risk Perception

Anticipated emotions were measured using approaches developed in pro-environmental behavior research (Joo et al., 2024). Environmental risk perception was measured using items adapted from the Ecological Cognition Scale (Bohlen et al., 1993) and the Risk Perception Scale (Mitchell & Boustani, 1994), as shown in Table 4.

### 3.2. Data Collection and Measurement Assessment

The survey was designed to collect data on social media use, environmental risk perception, anticipated emotions, and waste-sorting intentions. The questionnaire consisted of 24 items divided into five sections:

(1) screening questions to identify whether respondents had encountered waste-sorting information while using social media platforms (e.g., WeChat, Weibo, TikTok, Kuaishou);
(2) a social media use scale measuring information exposure and information gratification;
(3) an environmental risk perception scale measuring respondents’ awareness and perception of environmental risks;
(4) a waste-sorting intentions scale assessing respondents’ self-reported behavioral intentions;
(5) demographic questions collecting background information.

Except for the screening and demographic sections, all items were measured using a five-point Likert scale to ensure standardization and quantifiability. The full questionnaire is provided in Appendix A. Prior to the formal survey, a preliminary pre-survey was conducted to examine the measurement properties of the questionnaire. A total of 51 responses were collected. Four responses were excluded based on the following criteria:

(1) respondents selected “I have not encountered waste-sorting information while using social media platforms (e.g., WeChat, Weibo, TikTok, Kuaishou)” in the screening question;
(2) respondents failed to select “Somewhat Agree” for the attention-check (lie detector) item.

After data screening, 47 valid responses were retained for the pre-survey. Reliability- and validity-related statistics were calculated using SPSS 27.0, and the results are reported in Table 5.

Following the pre-survey, the formal questionnaire was distributed online via the Wenjuanxing platform using a convenience sampling approach. A total of 452 responses were collected. For the screening question regarding prior exposure to waste-sorting information on social media, 17 respondents selected “Never used”, and their questionnaires were terminated. For subsequent data analysis, responses were excluded if:

(1) respondents indicated no prior exposure to waste-sorting information on social media;
(2) respondents failed the attention-check item;
(3) or questionnaire completion time was less than one minute.

After applying these criteria, 435 valid questionnaires were retained for analysis. Descriptive statistics were calculated for the demographic characteristics of the sample, including gender, age, education level, occupation, living situation, city tier, and monthly income. The distribution of these variables is presented in Table 6.

Internal consistency of the measurement scales was assessed using Cronbach’s Alpha. In addition, average variance extracted (AVE) and composite reliability (CR) values were calculated. Sampling adequacy was examined using the Kaiser–Meyer–Olkin (KMO) measure, and Bartlett’s test of sphericity was conducted to assess inter-item correlations. The corresponding statistics are reported in Table 5 and Table 7.

### 3.3. Qualitative Interview Design and Analysis

In addition to the questionnaire survey, this study conducted semi-structured in-depth interviews as a supplementary qualitative component explicitly designed to support and interpret the quantitative findings. The interviews were not intended to test hypotheses or generate independent empirical claims, but rather to provide explanatory and contextual insights into the regression results, particularly by examining how individuals perceive waste-sorting information on social media, experience anticipated emotions, and interpret environmental risks in everyday contexts.

Building upon the regression analyses derived from the survey data, a total of 17 participants from different regions in China were interviewed. The interviewees included community managers, community residents, and students, allowing for the inclusion of diverse perspectives related to waste-sorting practices and social media use. Basic demographic information of the interview participants is presented in Table 8, and the interview outline is provided in Appendix B.

The interviews focused on participants’ subjective interpretations of waste-sorting-related content encountered on social media, their emotional reactions to such information, and perceived facilitators or constraints influencing their waste-sorting intentions. A semi-structured interview guide was used to ensure consistency across interviews while allowing respondents sufficient flexibility to elaborate on their personal experiences.

All interviews were audio-recorded with participants’ consent and transcribed verbatim for analysis. The interview data were analyzed using a thematic analysis approach. The analysis proceeded through three main stages: (1) familiarization with the interview transcripts through repeated reading; (2) identification of recurring concepts and patterns related to information perception, emotional responses, and behavioral intentions; and (3) iterative development of themes that were directly relevant to the interpretation of the quantitative mediation results.

Based on the results of the interview data, following findings are obtained.

All interviews were audio-recorded with participants’ consent and transcribed verbatim for analysis. The interview data were analyzed using a thematic analysis approach. The analysis proceeded through three stages:

(1) familiarization with the interview transcripts through repeated reading;
(2) identification of recurring concepts and patterns through coding;
(3) iterative development of themes.

## 4. Results

### 4.1. Regression Analysis Results

A detailed analysis of the relationships among social media use, including IE and IG, anticipated emotions, namely APE and ANE, ERP, and WSI was conducted, as summarized in Table 9. It can be noticed that social media IE shows a significant positive correlation with APE (r = 0.375, *p* < 0.01) and ANE (r = 0.455, *p* < 0.01). Similarly, social media IG is significantly positively correlated with APE (r = 0.434, *p* < 0.01) and ANE (r = 0.407, *p* < 0.01). APE (r = 0.466, *p* < 0.01) and ANE (r = 0.506, *p* < 0.01) both show strong positive correlations with WSI. Furthermore, higher levels of social media IE (r = 0.473, *p* < 0.01) and IG (r = 0.512, *p* < 0.01) are associated with stronger WSI among respondents. In addition, ERP is positively correlated with both APE (r = 0.458, *p* < 0.01) and ANE (r = 0.437, *p* < 0.01). These relationships highlight the role of environmental awareness in shaping emotional responses and subsequent behavioral intentions.

#### 4.1.1. Direct Effects Regression Analysis Results

The results of the direct effects regression analyses are presented in Table 10. First, with anticipated emotions as dependent variables, both IE and IG exhibit statistically significant associations with APE and ANE. Specifically, IG shows a stronger positive association with APE (β = 0.323, *p* < 0.001) than IE (β = 0.231, *p* < 0.001), whereas IE demonstrates a stronger positive association with ANE (β = 0.340, *p* < 0.001) than IG (β = 0.259, *p* < 0.001). These findings support H2a and H2b, indicating differentiated emotional activation effects of information gratification and information exposure. Second, when waste-sorting intentions are included as the dependent variable, both IE (β = 0.467, *p* < 0.001) and IG (β = 0.504, *p* < 0.001) show statistically significant positive effects on WSI. These results support H1a and H1b, suggesting that both dimensions of social media use are positively associated with waste-sorting intentions. Third, anticipated emotions are significantly associated with waste-sorting intentions. Specifically, APE (β = 0.456, *p* < 0.001) and ANE (β = 0.500, *p* < 0.001) both exhibit significant positive effects on WSI. Thus, H3a and H3b are supported. Detailed results are reported in Table 10.

#### 4.1.2. Mediating Effects Regression Analysis Results

This study employed the Bootstrap method to test the proposed mediating effects, and the results are presented in Table 11. The decomposition of indirect effects reveals three distinct mediating pathways, APE, ANE, and ERP, through which social media use influences waste-sorting intentions.

For IG, the total indirect effect on waste-sorting intentions is 0.263, accounting for 48.76% of the total effect. Specifically, the indirect effects via APE (effect = 0.069, 12.85%), ANE (effect = 0.069, 12.78%), and ERP (effect = 0.049, 9.12%) are all statistically significant, as the corresponding 95% confidence intervals do not include zero. These results indicate that both anticipated emotions and environmental risk perception serve as significant mediators between information gratification and waste-sorting intentions, thereby supporting H2a, H3a, H3b, H4b, and H5.

For IE, the total indirect effect on waste-sorting intentions is 0.280, accounting for 54.40% of the total effect. Among the specific mediating paths, the indirect effects through APE (effect = 0.073, 14.17%), ANE (effect = 0.085, 16.46%), and ERP (effect = 0.047, 9.10%) are all statistically significant. These findings indicate that anticipated emotions and environmental risk perception also mediate the relationship between information exposure and waste-sorting intentions, thus providing support for H2b, H3a, H3b, H4a, and H5.

Overall, the Bootstrap results confirm that both affective mediators (anticipated positive and negative emotions) and the cognitive mediator (environmental risk perception) play significant roles in linking social media use to waste-sorting intentions. Accordingly, the mediation-related hypotheses proposed in Section 2, namely H4a, H4b, and H5, are supported.

### 4.2. In-Depth Interviews Analysis Results

To further elucidate the mechanisms identified in the quantitative analyses, this study incorporates semi-structured in-depth interviews as a supplementary qualitative component. Guided by the theoretical framework and hypotheses developed in Section 2, and informed by the regression and mediation results reported in Section 4.1, the interview analysis focuses on three interrelated mechanisms: (1) the role of information exposure in activating anticipated negative emotions; (2) the role of information gratification in enhancing anticipated positive emotions; and (3) the mediating function of environmental risk perception linking social media use to waste-sorting intentions.

The quantitative results indicate that information exposure has a stronger predictive effect on anticipated negative emotions than information gratification, supporting H2b, and that information exposure is positively associated with environmental risk perception, supporting H4a. The interview data provide insights into how these relationships manifest in real-life communication settings. Many interviewees reported that waste-sorting-related information encountered on social media is predominantly conveyed through notices, reminders, and policy-oriented messages emphasizing obligations, compliance requirements, or potential consequences of non-compliance. Although such content increases information visibility, it often evokes feelings of pressure, concern, or guilt. As one community manager explained: “Much of the communication feels like task notifications, telling residents what they must do. If people fail to comply, they feel stressed rather than motivated.” (HYQ). Similarly, some residents described emotional fatigue when repeatedly exposed to messages highlighting responsibility and risk, especially when discrepancies between policy requirements and actual waste-handling practices were observed. These experiences help explain why higher levels of information exposure are more likely to elicit anticipated negative emotions, such as anxiety or regret, as identified in the regression results. At the same time, interviewees frequently referred to visual or narrative content depicting pollution, waste, and environmental degradation, which contributed to heightened awareness of environmental risks. Such accounts are consistent with the quantitative finding that information exposure significantly increases environmental risk perception, reflecting the risk amplification mechanism emphasized by the SARF.

In contrast, the quantitative analyses show that information gratification is a stronger predictor of anticipated positive emotions, providing support for H2a. The interview findings help clarify the psychological processes underlying this effect. When respondents perceived social media content as informative, comprehensible, and practically useful, particularly when it clarified waste-sorting procedures or explained policy rationales, they were more likely to report feelings of pride, satisfaction, or a sense of meaningful contribution. A student interviewee noted: “Some short videos explain waste sorting in a humorous way. After watching them, I feel it’s actually manageable and worth trying.” (ZSN). Such gratification-oriented content often reduces cognitive barriers and enhances individuals’ sense of competence, thereby activating positive anticipated emotions. Interviewees also emphasized the role of interactive and entertaining formats, such as short videos and memes, in fostering emotional engagement. These qualitative observations align with the quantitative evidence that information gratification more strongly predicts anticipated positive emotions than mere exposure.

Bootstrap mediation analyses indicate that environmental risk perception plays a significant but relatively modest mediating role between social media use and waste-sorting intentions, supporting H4a, H4b, and H5. The interview data provide further insight into why this cognitive pathway functions as a secondary mechanism. Interviewees commonly described environmental risk perception as a gradually accumulated awareness rather than an immediate trigger for behavior. Repeated exposure to environmental information on social media contributed to a general sense of concern about pollution and waste, but this concern alone was often insufficient to directly motivate waste-sorting behavior without supportive conditions. As one community resident remarked: “Most people know environmental pollution is serious, but whether they actually sort waste depends on convenience and whether others are doing it too.” (QSW). This perspective helps explain why environmental risk perception exhibits a statistically significant yet smaller indirect effect compared to affective pathways. Rather than directly driving behavior, risk perception appears to function as a cognitive background condition that reinforces emotional responses and contextualizes behavioral decisions.

Overall, the interview analysis complements the quantitative results by illustrating how distinct dimensions of social media use correspond to differentiated emotional and cognitive experiences in everyday contexts. Information exposure tends to heighten risk awareness and anticipated negative emotions, whereas information gratification is more closely associated with anticipated positive emotions through enhanced understanding and perceived efficacy. Environmental risk perception, in turn, operates as a supporting cognitive mechanism that amplifies but does not replace emotional pathways. By situating the statistical relationships within real-world communication practices, the qualitative evidence strengthens the interpretive validity of the proposed CAC–SARF integrated framework.

## 5. Discussion

### 5.1. Key Findings

This study examines how social media use shapes public waste-sorting intentions by integrating the CAC model with the SARF. Using a mixed-methods design, the findings identify a differentiated cognitive–affective mechanism through which social media influences pro-environmental behavioral intentions.

First, the results show that different dimensions of social media use activate distinct emotional pathways. Information gratification is more strongly associated with anticipated positive emotions, whereas information exposure exerts a stronger influence on anticipated negative emotions. Both anticipated positive and negative emotions significantly predict waste-sorting intentions. This finding is consistent with prior research emphasizing the importance of emotions in pro-environmental behavior (Bagozzi et al., 1999), while extending this literature by demonstrating that positive and negative anticipated emotions operate in parallel rather than in opposition.

Second, mediation analyses indicate that anticipated emotions and environmental risk perception jointly mediate the relationship between social media use and waste-sorting intentions, with emotional pathways accounting for a larger share of the indirect effects. The significant but comparatively weaker mediating role of environmental risk perception aligns with previous studies highlighting the role of risk awareness (Kollmuss & Agyeman, 2002; Zhang et al., 2025), while suggesting that cognitive risk perception alone may be insufficient to motivate behavioral intentions without affective engagement.

Third, qualitative interview evidence provides contextualized explanations for the quantitative results. Interview accounts indicate that fragmented and institution-centered information exposure tends to generate emotional pressure rather than sustained engagement, whereas content that is perceived as engaging, concrete, and practically informative is more likely to facilitate emotional involvement. These qualitative insights help explain the persistence of direct effects observed in the regression models and illustrate how the identified psychological mechanisms operate within real-world communication settings.

### 5.2. Theoretical Implications

This study offers several theoretical contributions to research on environmental communication and pro-environmental behavior.

First, by integrating CAC and SARF within a unified empirical framework, this study extends prior applications of both models by demonstrating how media-driven risk communication operates through affective mechanisms embedded in the cognition–affect–conation process. While CAC has traditionally been used to explain attitudinal and behavioral outcomes through sequential cognitive and emotional stages (Bagozzi et al., 1999), and SARF has emphasized the amplification of risk signals through media and social processes (Kasperson et al., 1988), the present findings show that risk-related information influences behavioral intentions primarily through emotional activation rather than through cognition alone. In this sense, the results align with and further refine recent extensions of SARF that emphasize individual-level psychological processing (Slovic, 2020).

Second, the findings contribute to affective theories of pro-environmental behavior by demonstrating that anticipated positive and negative emotions exert parallel motivational effects. This result aligns with prior studies suggesting that both reward-based emotions (e.g., pride, satisfaction) and avoidance-based emotions (e.g., guilt, regret) can motivate environmentally responsible intentions (Joo et al., 2024). However, unlike studies that emphasize the dominance of either positive or negative appeals, the present study shows that co-activation of mixed emotions constitutes a stable motivational configuration in social media environments characterized by high information density.

Third, the relatively weaker mediating role of environmental risk perception nuances existing applications of SARF by indicating that risk perception alone does not automatically translate into behavioral intention. This finding aligns with prior research documenting the intention–behavior gap in environmental contexts (Kollmuss & Agyeman, 2002) and extends it by showing that affective engagement functions as a necessary bridge between risk awareness and conative outcomes. In this regard, the study refines the theoretical assumption that amplified risk signals necessarily lead to action, highlighting instead the conditional role of emotional resonance.

Finally, the integration of qualitative insights strengthens the explanatory scope of the quantitative model by illuminating boundary conditions and residual mechanisms that are not fully captured by survey measures. Rather than generating independent theoretical claims, the qualitative evidence contextualizes how institutional communication styles and community environments shape the effectiveness of cognitive and affective pathways. This mixed-methods approach contributes to methodological discussions by demonstrating how qualitative data can be used to interpret mediation effects and clarify why statistically significant pathways may vary in strength across contexts.

### 5.3. Practical Implications

The findings offer several practical implications for policymakers, community administrators, and environmental communicators.

For public institutions and government agencies, the results suggest that waste-sorting campaigns should move beyond information-heavy and directive messaging strategies. While information exposure can raise awareness and concern, communication that better satisfies users’ informational and psychological needs, such as interactive formats, narrative storytelling, and emotionally engaging content, appears more effective in activating positive anticipated emotions and sustaining motivational engagement. Designing communication strategies that emphasize clarity, relevance, and emotional resonance may therefore enhance public responsiveness to waste-sorting initiatives.

For social media platforms and content producers, distinguishing between information exposure and information gratification is particularly important. High-frequency exposure may heighten concern or emotional pressure, whereas gratification-oriented content is more likely to foster emotionally grounded and stable behavioral intentions. Content strategies that balance informational clarity with emotional appeal may thus be more effective in promoting sustained pro-environmental participation.

At the community level, the findings highlight the importance of institutional credibility and infrastructural coherence. Even when residents exhibit strong emotional motivation and environmental concern, these psychological drivers may fail to translate into sustained behavior if waste-sorting systems are perceived as symbolic, fragmented, or procedurally inconsistent. Strengthening coordination across waste collection, transportation, and processing stages may help reinforce public trust and support the full cognition–affect–conation process identified in this study.

### 5.4. Limitations and Future Research

This study has several limitations that suggest avenues for future research. First, although institutional and structural constraints were identified through qualitative evidence, these factors were not directly quantified in the regression models. Future studies could employ multilevel modeling or incorporate objective community-level indicators to formally examine how institutional contexts moderate psychological pathways.

Second, the study focuses primarily on social media as an information source, potentially underestimating the influence of traditional media and offline community initiatives. Examining the interaction between online and offline communication environments would provide a more comprehensive understanding of how emotional and cognitive mechanisms develop over time.

Finally, the cross-sectional design limits causal inference regarding the temporal dynamics of emotional activation and risk perception. Longitudinal or experimental research designs could further clarify how repeated media exposure and emotional experiences accumulate to influence sustained waste-sorting behavior.

## Figures and Tables

**Table 1:** Key variables explanations.

Category	Concept	Operational Definition	Variables	Code
Dependent variable	waste-sorting intentions	The willingness of residents to participate in waste sorting.	Waste-Sorting Intentions	WSI
Independent variable	social media use	Residents’ media usage behavior through social media channels to access waste-sorting-related environmental information.	Information Exposure	IE
			Information Gratification	IG
Mediating variable	anticipated emotions	Positive or negative emotions individuals expect to experience when they engage or fail to engage in waste sorting.	Anticipated Positive Emotions	APE
			Anticipated Negative Emotions	ANE
	environmental risk perception	Intuitive judgments by individuals or social groups regarding highly complex and uncertain environmental issues.	environmental risk perception	ERP

**Table 2:** Measurement of the dependent variable.

Variable	Items
WSI	I strive to reduce the amount of waste I generate in daily life. If waste bins have clear classification labels, I would sort my household waste accordingly. If I fail to sort waste, I would voluntarily accept penalties (e.g., fines). I would try to teach my family, friends, or neighbors how to sort waste in daily life. I would attempt to participate in science education activities related to waste sorting. If possible, I would discourage improper waste sorting behaviors.

**Table 3:** Measurement of the independent variable.

Variable	Items
IE	I frequently see waste-sorting-related information on social media. Social media is my primary channel for accessing waste-sorting-related information. Using social media has become part of my daily routine. I discuss waste sorting with others (e.g., family, friends, classmates, or other netizens) on social media. When I don’t log into social media for a while, I feel disconnected from the world. I feel that I benefit from the dissemination of waste-sorting-related information on social media. I would regret it if social media lacked waste-sorting-related information.
IG	I acquire waste-sorting knowledge through social media. I find social media useful for learning about waste sorting. I can promptly access waste-sorting policies and regulations through social media. I learn about the relationship between waste sorting and environmental protection through social media. The government should actively use social media to promote waste sorting. Social media is an ethical way for the government to encourage public participation in waste sorting.

**Table 4:** Measurement of the mediating variable.

Variable	Items
APE	If I engage in waste-sorting behavior, I would feel proud. If I engage in waste-sorting behavior, I would feel happy. If I engage in waste-sorting behavior, I would feel it is meaningful.
ANE	If I fail to engage in waste-sorting behavior, I would feel regretful. If I fail to engage in waste-sorting behavior, I would feel angry. If I fail to engage in waste-sorting behavior, I would feel guilty.
ERP	I am aware that oceans and rivers are being polluted. I am aware that global warming is occurring. I am aware that tropical rainforests are diminishing. I am aware of air pollution caused by coal-fired power plants. I am aware of soil pollution caused by pesticide residues. I am aware of environmental pollution caused by insecticides.

**Table 5:** Reliability and validity testing of pre-survey scales.

Variable	Cronbach’s Alpha	KMO	Sig.	Number of Items
IE	0.661	0.604	<0.001	7
IG	0.863	0.691	<0.001	6
APE	0.85	0.705	<0.001	3
ANE	0.963	0.771	<0.001	3
ERP	0.943	0.843	<0.001	10
WSI	0.929	0.796	<0.001	6

**Table 6:** Demographic variables analysis (N = 435).

Variable	Category	Frequency	Percentage (%)
Gender	Male	180	41.4
	Female	255	58.6
Age	18 and below	37	8.5
	18–24	85	19.5
	25–35	152	34.9
	36–45	82	18.9
	46–60	65	14.9
	61 and above	14	3.2
Education	Middle school or below	26	6
	High school/vocational	67	15.4
	Associate degree	137	31.5
	Bachelor’s degree	172	39.5
	Graduate degree and above	33	7.6
Occupation	Government/Institution employee	70	16.1
	State/Private/Foreign enterprise employee	145	33.3
	Agriculture, forestry, livestock, fisheries	25	5.7
	Full-time student	89	20.5
	Freelancer	50	11.5
	Self-employed	22	5.1
	Unemployed/Homemaker	21	4.8
	Retired	9	2.1
	Others	4	0.9
Living Situation	Own a house	226	52
	Long-term rental	123	28.3
	Short-term rental	79	18.2
	Others	7	1.6
City Tier	Direct-controlled municipality	47	10.8
	Provincial capital	68	15.6
	Prefecture-level city	158	36.3
	County-level city	94	21.6
	Town	46	10.6
	Village	22	5.1
Monthly Income	Below 2000 yuan	57	13.1
	2000–5000 yuan	132	30.3
	5001–8000 yuan	165	37.9
	Above 8000 yuan	81	18.6

**Table 7:** Reliability coefficients of key variables (N = 435).

Variable	Cronbach’s Alpha	AVE (>0.5)	CR (>0.7)
IE	0.903	0.557	0.898
IG	0.907	0.579	0.892
APE	0.847	0.612	0.825
ANE	0.841	0.592	0.813
ERP	0.965	0.605	0.939
WSI	0.918	0.574	0.89

**Table 8:** Basic information of 17 participants.

	Participants	Age	Identity	City
1	HYQ	27	Community Manager	Nanning
2	LP	26	Community Manager	Liuzhou
3	ZJY	26	Community Manager	Nanning
4	ZSN	23	Student	Shenyang
5	SQ	30	Student	Beijing
6	HYX	33	Community Resident	Zhuhai
7	QSW	28	Community Resident	Shenzhen
8	HYX	32	Community Resident	Shanghai
9	TBW	26	Community Resident	Harbin
10	CJ	45	Community Manager	Nanjing
11	HYC	24	Student	Xi’an
12	LKR	25	Student	Kunming
13	CC	27	Community Resident	Beijing
14	XBT	24	Student	Hangzhou
15	HL	29	Community Manager	Xiamen
16	CT	45	Community Resident	Chongqing
17	HXY	38	Community Manager	Nanning

**Table 9:** Correlation analysis results of key variables.

Variable	IE	IG	APE	ANE	ERP	WSI
IE	1					
IG	0.611 **	1				
APE	0.375 **	0.434 **	1			
ANE	0.455 **	0.407 **	0.488 **	1		
ERP	0.412 **	0.472 **	0.458 **	0.437 **	1	
WSI	0.473 **	0.512 **	0.466 **	0.506 **	0.498 **	1

Note: ** *p* < 0.01.

**Table 10:** Regression analysis results of direct effects.

	Variables	Model 1	Model 2	Model 3	Model 4	Model 5	Model 6
Dependent variable		APE	ANE	WSI	WSI	WSI	WSI
Independent variables	IE	0.231 ***	0.34 ***			0.467 ***	
	IG	0.323 ***	0.259 ***				0.504 ***
	APE			0.456 ***			
	ANE				0.5 ***		
Control variables	Gender	Control	Control	Control	Control	Control	Control
	Age	Control	Control	Control	Control	Control	Control
	Education	Control	Control	Control	Control	Control	Control
	Occupation	Control	Control	Control	Control	Control	Control
	Living Situation	Control	Control	Control	Control	Control	Control
	City Tier	Control	Control	Control	Control	Control	Control
	Monthly Income	Control	Control	Control	Control	Control	Control
	F						
	R^2^	15.021	20.846	15.6	19.627	16.748	19.896
	Adjusted R^2^	0.241	0.372	0.227	0.269	0.239	0.272

Note: *** *p* < 0.001.

**Table 11:** Bootstrap test results for mediation effects.

Independent Variable	Impact Path	Effect Value	Standard Error	95% Confidence Interval		Relative Mediating Effect (%)
				Lower	Upper	
IG	Total Indirect Effect	0.263	0.029	0.207	0.321	48.76%
	Ind1: IG → APE → WSI	0.069	0.022	0.027	0.114	12.85%
	Ind2: IG → ANE → WSI	0.069	0.018	0.037	0.108	12.78%
	Ind3: IG → ERP → WSI	0.049	0.016	0.021	0.082	9.12%
IE	Total Indirect Effect	0.280	0.032	0.220	0.345	54.40%
	Ind6: IE → APE → WSI	0.073	0.021	0.034	0.117	14.17%
	Ind7: IE → ANE → WSI	0.085	0.021	0.047	0.131	16.46%
	Ind8: IE → ERP → WSI	0.047	0.016	0.019	0.081	9.10%

## Data Availability

The data used to support the findings of this study are available from the corresponding author upon request.

## Appendix A. Survey on the Relationship Between Social Media Use and Public Willingness to Engage in Waste Sorting

Dear Sir/Madam,

Thank you for taking the time to participate in this survey. This questionnaire aims to study the impact of social media use on the public’s willingness to engage in household waste sorting. The survey is completely anonymous, and no personal information will be disclosed. Your responses will be encoded for research analysis and used solely for academic purposes. Completing the questionnaire will take approximately 5–10 min. Please follow the instructions and answer truthfully. Your support is greatly appreciated!

General Knowledge and Experience with Waste Sorting

1. How well do you understand waste sorting? [Single choice]
  ○ Well-informed
  ○ Not informed
2. Have you ever practiced waste sorting? [Single choice]
  ○ Yes
  ○ No
3. Do you think waste sorting is necessary? [Single choice]
  ○ Necessary
  ○ Indifferent
  ○ Unnecessary
4. Have you encountered information about waste sorting while using social media (e.g., WeChat, Weibo, TikTok, Kuaishou)? [Single choice]
  ○ A. Yes
  ○ B. No (Please skip to the end of the questionnaire and submit your response)

Section 1: Social Media Usage

Please answer based on your social media usage habits. Select the option that best reflects your real situation. There are no right or wrong answers. Thank you for your participation!

5. How long have you been using social media? [Single choice]
  ○ Less than 1 year (started in 2023)
  ○ 1–2 years (started in 2022)
  ○ 2–3 years (started in 2021)
  ○ 3–4 years (started in 2020)
  ○ More than 4 years (started in 2019 or earlier)
6. How many hours do you use social media per week? [Single choice]
  ○ 0–6 h
  ○ 6–12 h
  ○ 12–18 h
  ○ 18–24 h
  ○ 24–30 h
  ○ More than 30 h
7. Exposure to waste sorting information on social media [Matrix scale]
  Please select the option that best reflects your experience.
  **Statement**	**Strongly Disagree**	**Disagree**	**Neutral**	**Agree**	**Strongly Agree**
  I often see waste sorting information on social media.	○	○	○	○	○
  Social media is my primary source for waste sorting information.	○	○	○	○	○
  Using social media has become part of my daily routine.	○	○	○	○	○
  I discuss waste sorting issues with others (e.g., family, friends, classmates, or online users) on social media.	○	○	○	○	○
  When I do not log in to social media for a while, I feel disconnected from the world.	○	○	○	○	○
  I feel that I benefit from waste sorting information shared on social media.	○	○	○	○	○
  I would feel regretful if social media lacked waste sorting information.	○	○	○	○	○
8. Satisfaction with waste sorting information on social media [Matrix scale]
  **Statement**	**Strongly Disagree**	**Disagree**	**Neutral**	**Agree**	**Strongly Agree**
  I have gained useful knowledge about waste sorting through social media.	○	○	○	○	○
  I believe social media is useful for learning about waste sorting.	○	○	○	○	○
  I can access waste sorting policies and regulations in a timely manner through social media.	○	○	○	○	○
  I have learned about the relationship between waste sorting and environmental protection through social media.	○	○	○	○	○
  The government should actively use social media to promote waste sorting.	○	○	○	○	○
  Social media is an ethical way for the government to encourage public participation in waste sorting.	○	○	○	○	○
9. Perception of waste sorting promotional content on social media [Matrix scale]
  **Statement**	**Strongly Disagree**	**Disagree**	**Neutral**	**Agree**	**Strongly Agree**
  Waste sorting promotional content is clear and easy to understand.	○	○	○	○	○
  The information is logically structured and well-organized.	○	○	○	○	○
  The content effectively uses appropriate text, images, and videos.	○	○	○	○	○
  The promotional language is engaging and humorous.	○	○	○	○	○
10. What types of waste sorting information have you encountered on social media? [Multiple choice]
  □ Static posters and infographics
  □ Policy-related articles
  □ Short videos, animations, environmental documentaries
11. How often do you pay attention to the following types of waste sorting information on social media? [Matrix scale]
  **Type of Information**	**Never**	**Rarely**	**Occasionally**	**Frequently**
  Positive waste sorting information	○	○	○	○
  Neutral waste sorting information	○	○	○	○
  Negative waste sorting information	○	○	○	○
  Section 2: Anticipated Emotions and Environmental Risk Perception
12. Please indicate your level of agreement with the following statements: [Matrix scale]
  **Statement**	**Strongly Disagree**	**Disagree**	**Neutral**	**Agree**	**Strongly Agree**
  I am aware that oceans and rivers are being polluted.	○	○	○	○	○
  I am aware that global warming is happening.	○	○	○	○	○
  I am aware that tropical rainforests are decreasing.	○	○	○	○	○
  I am aware that coal power plants contribute to air pollution.	○	○	○	○	○
13. How do you feel about engaging in waste sorting? [Matrix scale]
  **Statement**	**Strongly Disagree**	**Disagree**	**Neutral**	**Agree**	**Strongly Agree**
  I feel proud when I engage in waste sorting.	○	○	○	○	○
  Waste sorting makes me feel happy.	○	○	○	○	○
  Waste sorting feels meaningful.	○	○	○	○	○
  If I do not engage in waste sorting, I feel guilty.	○	○	○	○	○
  Section 3: Policy Effectiveness and Willingness to Engage in Waste Sorting
14. Please indicate your level of agreement with the following statements: [Matrix scale]
  **Statement**	**Strongly Disagree**	**Disagree**	**Neutral**	**Agree**	**Strongly Agree**
  I am more willing to sort waste if there are material incentives.	○	○	○	○	○
  Improved environmental infrastructure encourages me to practice waste sorting.	○	○	○	○	○
  Demographic Information
15. Educational Level: [Single choice]
  ○ Junior high school or below
  ○ High school/Vocational school
  ○ Associate degree
  ○ Bachelor’s degree
  ○ Master’s degree or above
16. Employment Status: [Single choice]
  ○ Government/Public Sector
  ○ Enterprise Employee
  ○ Student
  ○ Self-employed
  ○ Retired
  ○ Other: _________
17. Monthly Income: [Single choice]
  ○ Below 2000 RMB
  ○ 2000–5000 RMB
  ○ 5001–8000 RMB
  ○ Above 8000 RMB
  Thank you for your time and participation!

## Appendix B. Interview Outline on the Impact of Social Media Use on Residents’ Willingness to Engage in Waste Sorting

1. Community Residents Interview Outline
  - What are your thoughts on the frequency and content of community activities related to waste sorting?
  - How do you feel about the current effectiveness of waste sorting in your community?
  - What are the reasons for your participation or non-participation in these community activities?
  - Under what circumstances would you be willing to engage in community governance or activities?
  - Have any of the activities you participated in left a deep impression on you? Why?
  - After participating in these activities, have you or your family experienced any changes?
  - Are there any community regulations that encourage residents to participate in community activities?
  - Are you familiar with the community staff responsible for promoting waste sorting? Are you satisfied with their services?
  - In community waste sorting campaigns, do you prefer traditional media, social media, or collective community events as a promotion method?
  - What issues do you think still exist in waste sorting projects within community environmental management?
  - What suggestions do you have for these projects and activities?
2. Functional Department Staff Interview Outline
  - Is the community currently implementing a waste sorting program?
  - What is the role of the residents’ committee in waste sorting efforts?
  - How do residents spontaneously participate in waste sorting?
  - What are the main difficulties preventing residents from participating in waste sorting?
  - How are waste sorting bins set up within the community?
  - Are household waste materials properly sorted and collected for transport? How is this process managed?
  - Is there any regulatory oversight in the process of promoting waste sorting? How strong is the enforcement?
